# Mapping of the binding sites of human diamine oxidase (DAO) monoclonal antibodies

**DOI:** 10.1007/s00011-017-1118-3

**Published:** 2017-11-21

**Authors:** Hubert G. Schwelberger, Johannes Feurle, Gunnar Houen

**Affiliations:** 1 Department of Visceral, Transplant and Thoracic Surgery, Molecular Biology Laboratory, Medical University Innsbruck, Schöpfstraße 41, 6020 Innsbruck, Austria; 2 Department of Autoimmunology and Biomarkers, Statens Serum Institut, Artillerivej 5, 2300 Copenhagen, Denmark

**Keywords:** Diamine oxidase, Histamine metabolism, Monoclonal antibodies, Protein expression, Epitope mapping

## Abstract

**Objective:**

Recently we characterized five mouse monoclonal antibodies that allow the specific and sensitive detection of human diamine oxidase (DAO). To understand differences in binding characteristics and recognition of enzyme variants, we mapped the antibody binding sites.

**Methods:**

Fragments of human DAO were expressed as glutathione-*S*-transferase fusion proteins that were used for testing antibody binding on immunoblots. Combined information from species cross-reactivity, sequence comparison and binding site-prediction software were used to localize the epitope recognized by each antibody.

**Results:**

All five monoclonal DAO antibodies bound to linear epitopes between the N3 and enzymatic domains of the 732 amino acid protein. The binding sites could be mapped onto amino acid regions V^262^-E^278^ and P^279^-R^288^, respectively, which exhibit considerable sequence variation in mammals explaining the fact that the human DAO antibodies do not cross-react with DAO from other species. The antibodies efficiently bind only denatured human DAO but not the native protein.

**Conclusions:**

Characterization of the binding sites of the DAO antibodies revealed that the antibodies bind two adjacent epitopes and exhibit similar binding characteristics and species cross-reactivity. As the epitopes do not overlap any of the amino acid substitutions described for clinically significant DAO gene polymorphisms, our antibodies will also be useful for analyses of the mutant DAO proteins.

## Introduction

Histamine mediates many biological processes including inflammation, gastric acid secretion, neuromodulation, and regulation of immune function by binding and activating four different G-protein-coupled receptors [1, 2]. Histamine has potent activity at low concentrations and its synthesis, transport, storage, release, and degradation have to be tightly regulated to avoid undesirable reactions. Histamine is synthesized by decarboxylation of the amino acid L-histidine, catalysed by the enzyme histidine decarboxylase (HDC, EC 4.1.1.22) [3, 4]. In mammals, histamine can be inactivated either by methylation of the imidazole ring nitrogen, catalysed by histamine *N*-methyltransferase (HNMT, EC 2.1.1.8) or by oxidative deamination of the primary amino group, catalysed by diamine oxidase (DAO, EC 1.4.3.22) [4–6].

Human DAO is a homodimeric glycoprotein of ca. 200 kDa consisting of two polypeptide chains of 732 amino acid residues [4, 5]. DAO uses molecular oxygen to oxidize the primary amino group of histamine forming imidazole acetaldehyde, ammonia, and hydrogen peroxide [4, 5]. Besides histamine, DAO efficiently converts numerous other di- and polyamines including putrescine, cadaverine, agmatine, and spermidine [5, 7]. The human protein is encoded by a single gene designated AOC1 that has five exons and has been mapped to chromosome 7q36.1 [8–10]. DAO is a secretory protein with an N-terminal signal peptide and has three copper amine oxidase domains [4, 11].

In order to characterize the human histamine inactivating enzymes, we recently produced and characterized a series of mouse monoclonal antibodies specific for DAO and HNMT, respectively [12, 13]. These antibodies turned out to be invaluable tools for the study of the expression and cellular localization of the enzymes. In contrast to polyclonal antibodies produced against porcine DAO that bound DAO proteins also from various other species [14], the five monoclonal antibodies binding human DAO did not show any cross-reactivity with non-human DAO [12]. The monoclonal antibodies showed excellent specificity and sensitivity for the detection of normal human DAO. Yet, we asked if they could recognize and be used to detect enzyme variants that have been described resulting from single nucleotide polymorphisms (SNPs) of the AOC1 gene and that might be relevant for diseases associated with impaired histamine inactivation [15–17]. Therefore, we tested antibody binding to various fragments of the DAO protein expressed in vitro and combined these results with data from sequence comparison, species cross-reactivity, structural information, and binding site-prediction tools to map the epitopes recognized by the DAO antibodies.

## Materials and methods

### Preparation and expression of recombinant human DAO protein fragments

Full-length human DAO cDNA [8, 9] was amplified by PCR with specific primers from total human placenta cDNA and cloned in frame into the *Eco*RI site of the bacterial expression vector pGEX-5X-1 (GE Healthcare, Vienna, Austria) to obtain plasmid pGEX-huDAO. Expression plasmids pGEX-huDAO01-04 were obtained by subcloning of the *Sma*I^66^-*Sma*I^566^ fragment (encoding E^23^-R^190^), the *Sma*I^566^-*Dra*I^1303^ fragment (encoding R^190^-F^435^), the *Dra*I^1303^-*Eco*RI^2255^ fragment (encoding K^436^-V^751^), and the *Sma*I^566^-*Eco*RI^2255^ fragment (encoding R^190^-V^751^), respectively, in frame into the *Sma*I or *Sma*I-*Eco*RI sites of pGEX-5X-1/-2/-3 (superscripts indicate position of recognition sequence relative to A^1^ of translational start codon or amino acid position relative to M^1^ of precursor protein, respectively) [12].

A series of C-terminal deletions of the *Sma*I^566^-*Dra*I^1303^ fragment cloned in pGEX-huDAO02 was produced by double-digestion with restriction endonucleases *Sma*I^566^ and *Sac*I^652^, *Sac*II^861^, *Apa*LI^1015^, or *Tsp*45I^1141^, creating blunt ends by incubation for 15 min at 37 °C with 1 U Klenow Fragment and 100 µM dNTPs, and inserting the fragments in frame into the *Sma*I sites of pGEX-5X-3, resulting in plasmids pGEX-huDAO11-14. A series of *Hae*III fragments of the *Sma*I^566^-*Dra*I^1303^ fragment cloned in pGEX-DAO02 was produced by digestion with restriction endonuclease *Hae*III and inserting the *Hae*III^583^-*Hae*III^693^, *Hae*III^693^-*Hae*III^895^, *Hae*III^919^-*Hae*III^1129^, and *Hae*III^1129^-*Dra*I^1303^ fragments in frame into the *Sma*I sites of pGEX-5X-1/-3, resulting in plasmids pGEX-huDAO21-24.

**Fig. 1:**
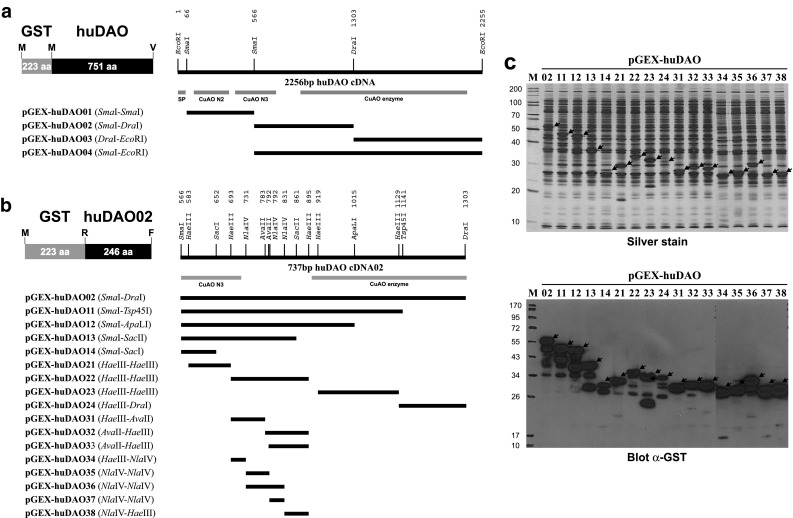
DAO expression constructs. **a** Recombinant plasmids expressing GST-DAO fusion proteins used for immunization that were obtained by cloning different human DAO cDNA fragments into the bacterial expression vectors pGEX-5X-1/-2/-3, respectively. Grey bars indicate the positions of the copper amine oxidase N-terminal domains 2 and 3 (CuAO N2/N3), the enzymatic core domain (CuAO enzyme), and the signal peptide sequence (SP). **b** Recombinant plasmids expressing GST-DAO fusion proteins obtained by subcloning cDNA fragments from pGEX-huDAO02 into the bacterial expression vectors pGEX-5X-1/-2/-3, respectively. **c** 12.5% Silver-stained SDS polyacrylamide gel and immunoblot with an anti-GST antibody of lysates prepared from bacteria-harbouring plasmids pGEX-huDAO02-38 indicated on top of each lane. Migration positions of full-length fusion proteins are indicated by arrows, and their expected sizes are listed in Table 1. The sizes of molecular weight markers (M) are given on the left in kDa

The *Hae*III^693^-*Hae*III^895^ fragment cloned in pGEX-huDAO22 was digested partially with *Ava*II, blunt ends were created with Klenow Fragment, and fragments *Hae*III^693^-*Ava*II^783^, *Ava*II^783^-*Hae*III^895^, and *Ava*II^792^-*Hae*III^895^ were cloned in frame into the *Sma*I site of pGEX-5X-3/-2 resulting in plasmids pGEX-huDAO31-33. The *Hae*III^693^-*Hae*III^895^ fragment was also digested with *Nla*IV and fragments *Hae*III^693^-*Nla*IV^731^, *Nla*IV^731^-*Nla*IV^792^, *Nla*IV^731^-*Nla*IV^831^, *Nla*IV^792^-*Nla*IV^831^, and *Nla*IV^831^-*Hae*III^895^ were inserted in frame into the *Sma*I site of pGEX-5X-3/-1 resulting in plasmids pGEX-huDAO34-38. All cloning enzymes were obtained from Thermo Scientific (Vienna, Austria). The clones were checked by DNA sequence analyses and their inserts and the resulting DAO protein fragments are illustrated in Fig. 1a, b and detailed in Table 1.

Each recombinant plasmid was transformed into the protease-deficient strain *E. coli* BL21 to produce glutathione *S*-transferase (GST) fusion proteins according to manufacturer’s instructions (GE Healthcare, Vienna, Austria). Briefly, recombinant bacteria were grown at 37 °C with slight agitation (100 rpm) in 10 ml YTA (16 g/l tryptone, 10 g/l yeast extract, 5 g/l NaCl, 100 mg/l ampicillin, pH 7.0) to an OD_600nm_ of 0.5 and fusion protein expression was induced for 4 h by addition of 0.1 mM isopropyl-β-d-thiogalactopyranoside (IPTG, Roche, Vienna, Austria). Bacteria were harvested by centrifugation for 5 min at 4000×*g*, 4 °C, washed with cold deionized water, and lysed in SDS sample buffer by incubation for 10 min at 95 °C. Cell lysates were cleared by centrifugation for 5 min at 10000×*g* and stored at − 20 °C until use. Fusion protein expression was analysed by SDS polyacrylamide gel electrophoresis [18] and Western blotting [19] using the GST-specific monoclonal antibody HYB374-01, which showed considerable expression for all constructs (Fig. 1c).

**Table 1:** Expression plasmids for huDAO fragments

Plasmid	Vector	cDNA fragment	Peptide	FuP (kDa)
pGEX-huDAO01	pGEX-5X-1	*Sma*I^66^-*Sma*I^566^	P^23^-R^190^	44.5
pGEX-huDAO02	pGEX-5X-3	*Sma*I^566^-*Dra*I^1303^	R^190^-F^435^	53.1
pGEX-huDAO03	pGEX-5X-2	*Dra*I^1303^-*Eco*RI^2255^	K^436^-V^751^	60.8
pGEX-huDAO04	pGEX-5X-3	*Sma*I^566^-*Eco*RI^2255^	R^190^-V^751^	87.8
pGEX-huDAO11	pGEX-5X-3	*Sma*I^566^-*Tsp*45I^1141^	R^190^-V^381^	47.1
pGEX-huDAO12	pGEX-5X-3	*Sma*I^566^-*Apa*LI^1015^	R^190^-V^339^	42.5
pGEX-huDAO13	pGEX-5X-3	*Sma*I^566^-*Sac*II^861^	R^190^-R^288^	36.9
pGEX-huDAO14	pGEX-5X-3	*Sma*I^566^-*Sac*I^652^	R^190^-E^218^	29.2
pGEX-huDAO21	pGEX-5X-1	*Hae*III^583^-*Hae*III^693^	Q^196^-W^231^	30.0
pGEX-huDAO22	pGEX-5X-3	*Hae*III^693^-*Hae*III^895^	V^233^-G^299^	33.4
pGEX-huDAO23	pGEX-5X-1	*Hae*III^919^-*Hae*III^1129^	P^308^-G^377^	33.7
pGEX-huDAO24	pGEX-5X-1	*Hae*III^1129^-*Dra*I^1303^	L^378^-F^435^	32.4
pGEX-huDAO31	pGEX-5X-3	*Hae*III^693^-*Ava*II^783^	V^233^-V^262^	29.3
pGEX-huDAO32	pGEX-5X-2	*Ava*II^783^-*Hae*III^895^	V^262^-G^299^	30.2
pGEX-huDAO33	pGEX-5X-2	*Ava*II^792^-*Hae*III^895^	D^265^-G^299^	29.9
pGEX-huDAO34	pGEX-5X-3	*Hae*III^693^-*Nla*IV^731^	V^233^-G^244^	27.3
pGEX-huDAO35	pGEX-5X-3	*Nla*IV^731^-*Nla*IV^792^	P^246^-E^264^	28.1
pGEX-huDAO36	pGEX-5X-3	*Nla*IV^731^-*Nla*IV^831^	P^246^-E^278^	29.6
pGEX-huDAO37	pGEX-5X-1	*Nla*IV^792^-*Nla*IV^831^	P^266^-E^278^	27.4
pGEX-huDAO38	pGEX-5X-1	*Nla*IV^831^-*Hae*III^895^	P^279^-G^299^	28.3

Human DAO cDNA fragments obtained with different restriction endonucleases were cloned in frame into the expression vectors pGEX-5X-1/-2/-3 to produce different size GST-DAO fusion proteins (FuP). Superscripts indicate position of restriction site on cDNA sequence (relative to A^1^ of translational start codon) and amino acid position (relative to M^1^ of precursor protein), respectively

### Testing of the binding of DAO-specific monoclonal antibodies

Monoclonal antibodies HYB313-01/-02/-03/-04 and HYB311-01 specific for human DAO [12] were tested for binding to different DAO fragments using filter strips of cell lysates containing the expressed GST fusion proteins. Cleared cell lysates prepared in SDS sample buffer containing approximately 100 µg protein were separated on 12.5% SDS polyacrylamide gels [18] and blotted onto polyvinylidene fluoride (PVDF) membranes [19]. After washing in TBST (50 mM Tris.HCl, pH 7.5, 150 mM NaCl, 0.1% Tween 20) and blocking non-specific binding sites by incubation for 60 min at 4 °C in TBSTM (TBST containing 2% non-fat dry milk) the membranes were cut into 20 vertical filter strips each containing circa 5 µg of protein. Each filter strip was incubated for 16 h at 4 °C with suitable dilutions of the monoclonal antibodies in TBSTM, washed 4 × 5 min with TBST, incubated 60 min at 4 °C with horseradish peroxidase-conjugated anti-mouse immunoglobulins (Dako, Glostrup, Denmark) diluted 1:1500 in TBSTM, washed 4 × 5 min with TBST, incubated 5 min with ECL reagent (GE Healthcare, Vienna, Austria), and exposed to Cronex 5 film (Agfa, Mortsel, Belgium).

Binding specificity was further tested by inhibition of antibody binding to DAO by soluble fusion proteins. The fusion proteins encoded by plasmids pGEX-huDAO22/32/33/36/38 were expressed as described above and soluble bacterial extracts were prepared by centrifugation for 5 min at 20000×*g* after lysing bacteria with BugBuster Protein Extraction Reagent containing 25 U/ml Benzonase and 1000 U/ml rLysozyme (Merck, Darmstadt, Germany). Antibodies HYB313-01/-02/-03/-04 and HYB311-01 were pre-incubated for 20 min at 4 °C with slight agitation in TBST with a ca. 2-, 20-, and 200-fold molar excess of the respective fusion protein and then incubated for 16 h at 4 °C with filter strips containing ca. 5 µg human seminal plasma protein with considerable amounts of DAO [20]. Following incubation with horseradish peroxidase-conjugated anti-mouse immunoglobulins, blots were developed with ECL reagent and exposed to film as described above. Signal intensity was compared with controls, where either no fusion protein or a ca. 2-, 20-, and 200-fold molar excess of GST expressed from pGEX-5X-1 had been added during pre-incubation.

Immunoprecipitation experiments were carried out to test if the antibodies also bind the native DAO protein. Total lysates were prepared from human and porcine kidney, respectively, by homogenization of ca. 50 mg tissue in 1 ml lysis buffer (20 mM bis.Tris.HCl, pH 7.0, 5 mM dithiothreitol) containing Complete Protease Inhibitor Cocktail (Roche, Vienna, Austria) for 5 min at 30 Hz using a TissueLyser II homogenizer (Qiagen, Hilden, Germany). Lysates were cleared by centrifugation for 10 min at 20000×*g*, 4 °C and the supernatant containing the total soluble protein was stored at − 20 °C until used. Lysates containing comparable DAO activity were incubated with slight agitation in a total volume of 100 µl with different concentrations of the monoclonal DAO antibodies for 16 h at 4 °C, followed by incubation with Protein A-Sepharose (GE Healthcare, Vienna, Austria) for 1 h at 4 °C. Immunoprecipitates were separated by centrifugation for 1 min at 6700×*g*, 4 °C, washed 3 times with TBST, and solubilized in SDS sample buffer. The presence of DAO was analysed in the precipitate and in the supernatant by immunoblotting with HYB313-03 and DAO activity was determined in the supernatant by a radiometric assay with [1,4-^14^C]putrescine dihydrochloride (GE Healthcare, Vienna, Austria) as the substrate as described previously [21]. Precipitation with a non-specific monoclonal antibody served as control.

### Mapping of binding sites using binding region information, sequence comparison, and antibody cross-reactivity

DAO polypeptide sequences were aligned using the NCBI Constrained-based Multiple Alignment Tool (http://www.ncbi.nlm.nih.gov/tools/cobalt/cobalt.cgi?link_loc=BlastHomeLink) [22]. Antigenicity plots were produced with the BepiPred Linear Epitope Prediction Tool (tools.immuneepitope.org/bcell) [23]. For testing species cross-reactivity, filter strips prepared from cleared tissue lysates of human and porcine kidney and of rat and mouse intestine were incubated with the different DAO antibodies and developed as described above. For detection of weak bands, ECL Prime reagent (GE Healthcare, Vienna, Austria) was substituted for ECL reagent. Structural views were created with the NCBI Cn3D 4.3.1 software [24] using DAO structure 3HI7 [11].

## Results

**Fig. 2:**
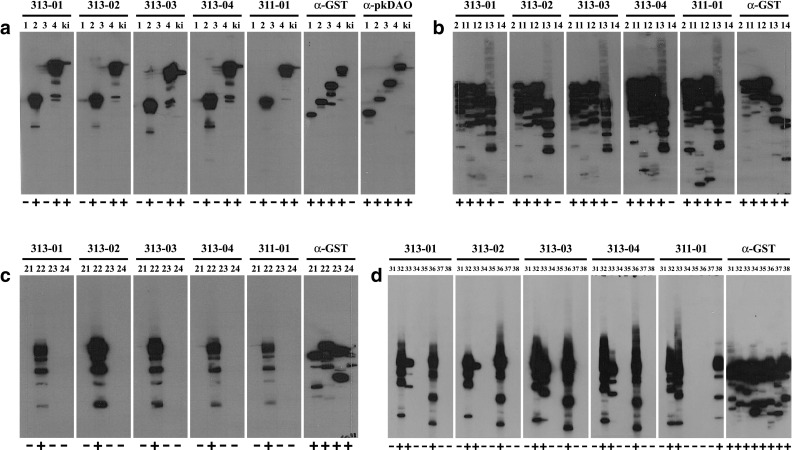
Antibody binding to DAO fragments. Antibody binding was tested using filter strips of DAO fragments expressed from pGEX-huDAO01-04 used for immunization (**a**), of DAO fragments expressed from pGEX-huDAO11-14 with C-terminal deletions of pGEX-huDAO02 (**b**), of DAO fragments expressed from pGEX-huDAO21-24 with *Hae*III fragments of pGEX-huDAO02 (**c**), or of DAO fragments expressed from pGEX-huDAO31-38 obtained by subcloning of pGEX-huDAO22 (**d**). Filter strips containing approximately 5 µg cell lysate protein separated on a 12.5% SDS polyacrylamide gel were incubated with the mouse monoclonal antibodies HYB313-01/-02/-03/-04, HYB311-01 (diluted 1:7500–1:20,000 in TBSTM), the anti-GST antibody HYB374-01 (1:1500), or the rabbit polyclonal antibody α-pkDAO (1:10,000) made against porcine kidney DAO, respectively. Filter strips were then incubated with horseradish peroxidase-conjugated anti-mouse immunoglobulins (1:1500 in TBSTM) or anti-rabbit immunoglobulins (1:5000), respectively, followed by ECL substrate, and exposure to film for 0.25–10 min. Lane numbers 1–4, 11–14, 21–24, and 31–38 correspond to expression constructs pGEX-huDAO01-04, pGEX-huDAO11-14, pGEX-huDAO21-24, and pGEX-huDAO31-38, respectively, and ki to a human kidney lysate. Binding is indicated by + and − signs below each lane. Exact positions of the bands on parallel lanes vary slightly because filter strips from different individual blots were used for this experiment. Besides the major band of the respective full-length fusion protein, a variable number of smaller bands is visible in most lanes due to production of partial products in the bacterial expression system

Using human DAO cDNA fragments expressed in vitro as antigens, we recently produced a series of mouse monoclonal antibodies that bind to human DAO and facilitate the specific and sensitive detection of the protein on immunoblots of human lysates and by immunohistochemical staining of human tissues [12]. Antibody clones HYB313-01/-02/-03/-04 resulted from immunization with DAO fragment R^190^-F^435^ expressed by pGEX-huDAO02, and clone HYB311-01 resulted from immunization with DAO fragment R^190^-V^751^ expressed by pGEX-huDAO04 (Fig. 1a; Table 1). Whereas a polyclonal antibody produced earlier against porcine DAO [14] bound to all four constructs expressed by pGEX-huDAO01-04, the five monoclonal human DAO antibodies bound only to the fusion proteins expressed by pGEX-huDAO02 and pGEX-huDAO04 (Fig. 2a), indicating that their binding sites are located on peptide R^190^-F^435^ expressed by pGEX-huDAO02.

In order to analyse where on this fragment the antibodies bind, a series of C-terminal deletions of peptide R^190^-F^435^ were constructed by recombinant DNA technology, and the resulting polypeptides were expressed as GST fusions in bacteria (Fig. 1b; Table 1). Bacterial lysates containing considerable amounts of the respective fusion proteins (Fig. 1c) were then separated by SDS polyacrylamide gel electrophoresis and blotted onto PVDF membranes to test the binding of the antibodies. As shown in Fig. 2b, all five antibodies gave strong signals with the fusion proteins expressed by plasmids pGEX-huDAO11/12/13 but not pGEX-huDAO14, indicating that their binding requires residues downstream of *Sac*I^652^ or E^218^ and upstream of *Sac*II^861^ or R^288^, respectively.

In order to confirm that binding occurs in this region, a series of *Hae*III fragments was produced from the cDNA cloned in pGEX-huDAO02 (Fig. 1b; Table 1), and the resulting fusion proteins were used for testing antibody binding. As shown in Fig. 2c, all five antibodies bound only to peptide V^233^-G^299^ encoded by the *Hae*III^693^-*Hae*III^895^ fragment expressed by pGEX-huDAO22. Combined with the information from the binding experiments with the C-terminal deletions, it could be concluded that all antibodies should bind in the region *Hae*III^693^-*Sac*II^861^ or V^233^-R^288^, respectively.

To more precisely localize the binding sites, the *Hae*III^693^-*Hae*III^895^ fragment expressed by pGEX-huDAO22 was further subcloned after restriction with *Ava*II and *Hae*III, respectively (Fig. 1b; Table 1). As shown in Fig. 2d, the antibodies did not bind to the fusion protein expressed by pGEX-huDAO31 but all strongly bound to the fusion protein expressed by pGEX-huDAO32, indicating that their binding sites are downstream of *Ava*II^783^ or V^262^, respectively. Whereas HYB313-03 and HYB311-01 also strongly bound to peptide D^265^-G^299^ encoded by the *Ava*II^792^-*Hae*III^895^ fragment of pGEX-huDAO33, HYB313-01/-02 exhibited significantly weaker and HYB313-04 slightly weaker binding to this fragment, indicating that strong binding of these antibodies also requires residues V^262^-E^264^.

When testing binding of the antibodies to the smaller *Nla*IV fragments made from the *Hae*III^693^-*Hae*III^895^ region (Fig. 1b; Table 1), it was found HYB313-01/-02/-03/-04 did not bind to any of the fusion proteins expressed by pGEX-huDAO34/35/37/38 containing the non-overlapping fragments *Hae*III^693^-*Nla*IV^731^, *Nla*IV^731^-*Nla*IV^792^, *Nla*IV^792^-*Nla*IV^831^, and *Nla*IV^831^-*Hae*III^895^, respectively, but only to the fusion expressed by pGEX-huDAO36 containing fragment *Nla*IV^731^-*Nla*IV^831^ that combines fragments *Nla*IV^731^-*Nla*IV^792^ and *Nla*IV^792^-*Nla*IV^831^ (Fig. 2d). Therefore, apparently the binding sites of these antibodies are interrupted by *Nla*IV^792^ and are upstream of *Nla*IV^831^ or E^278^, respectively. In contrast, HYB311-01 bound to the fusion protein expressed by pGEX-huDAO38, indicating that its binding site is downstream of *Nla*IV^831^ or P^279^, respectively.

Binding specificity was confirmed by inhibition of antibody binding on blots of human DAO present in human seminal plasma [20] by pre-incubation of antibodies with soluble fusion proteins. As shown in Table 2, binding of all antibodies was strongly inhibited by pre-incubation with the fusion proteins expressed from pGEX-huDAO22 and pGEX-huDAO32 encoding peptides V^233^-G^299^ and V^262^-G^299^, respectively, and a ca. 20-fold molar excess was sufficient to completely block binding. Whereas peptide D^265^-G^299^ expressed from pGEX-huDAO33 inhibited binding of HYB313-03 and HYB311-01 as efficiently as peptide V^262^-G^299^, it only partially inhibited binding of HYB313-01/-04 and did not inhibit binding of HYB313-02 at all, indicating that residues V^262^-E^264^ are required for efficient binding of HYB313-01/-02/-04. In accordance with the direct binding experiments described above, the fusion protein containing peptide P^246^-E^278^ expressed from pGEX-huDAO36 strongly inhibited binding of HYB313-01/-02/-03/-04 but did not affect binding of HYB311-01, whereas peptide P^279^-G^299^ expressed from pGEX-huDAO38 strongly inhibited binding of HYB311-01 but did not affect binding of HYB313-01/-02/-03/-04.

**Table 2:** Inhibition of antibody binding by soluble fusion proteins

Plasmid	Fusion protein	HYB313-01	HYB313-02	HYB313-03	HYB313-04	HYB311-01
pGEX-huDAO22	GST-V^233^-G^299^	++	++	++	++	++
pGEX-huDAO32	GST-V^262^-G^299^	++	++	++	++	++
pGEX-huDAO33	GST-D^265^-G^299^	+	−	++	+	++
pGEX-huDAO36	GST-P^246^-E^278^	++	++	++	++	−
pGEX-huDAO38	GST-P^279^-G^299^	−	−	−	−	++
pGEX-5X-1	GST	−	−	−	−	−

Inhibition of antibody binding was tested on blots of human seminal plasma that contains considerable amounts of DAO protein [20]. Antibodies HYB313-01/-02/-03/-04 and HYB311-01 were pre-incubated with a 2–200-fold molar excess of the fusion proteins expressed from plasmids pGEX-huDAO22/32/33/36/38 or pGEX-5X-1 and then incubated with blots of human seminal plasma followed by incubation with horseradish peroxidase-conjugated anti-mouse immunoglobulins. Blots were developed with ECL substrate, exposed to film, and signal intensity was compared with controls lacking fusion protein during pre-incubation. ++ strong/+ weak/− no inhibition of binding

**Fig. 3:**
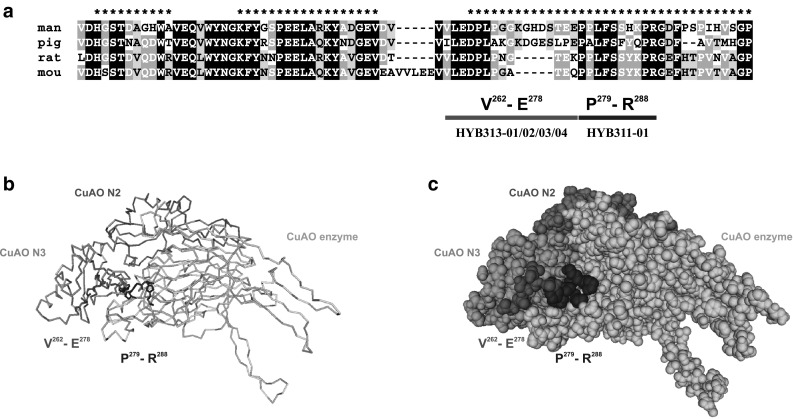
Localization of antibody binding sites. **a** Alignment of human, pig, rat, and mouse DAO polypeptide sequences of the region V^221^-P^300^ with predicted linear epitopes indicated by * on top [23]. Residues identical in all four proteins are shaded black, residues identical in three proteins are shaded dark grey, and residues identical in two proteins are shaded light grey, respectively. Peptide fragments V^262^-E^278^ and P^279^-R^288^ binding antibodies HYB313-01/-02/-03/-04 and HYB311-01, respectively, are indicated below the sequence as red and blue bars. **b** Protein backbone model and **c** space filling model of chain A of human DAO structure 3HI7 [11] with copper amine oxidase domains shown in different shades of grey and antibody binding peptides V^262^-E^278^ and P^279^-R^288^ in red and blue, respectively. Peptide V^262^-E^278^ (red) is only partially visible because residues P^268^-E^277^ have not been modelled in this structure due to weak uninterpretable electron density resulting from local disorder (A. McGrath, personal communication). Chain B of the homodimeric DAO protein was omitted for clarity and would be localized symmetrically on the south-eastern side of this image. (Color figure online)

Taken together, the binding sites of all antibodies could be localized in the small region V^262^-R^288^ between the CuAO N3 and enzymatic domains of DAO and appeared to be linear epitopes. As illustrated in Fig. 3, HYB313-01/-02/-03/-04 bound in region V^262^-E^278^ and HYB311-01 in region P^279^-R^288^ of human DAO. Using blots of tissue homogenates containing comparable DAO activity to test species cross-reactivity, all five antibodies bound only to human DAO but not to DAO proteins from pig, rat, or mouse (Table 3). This could be explained by the fact that the region corresponding to V^262^-E^278^ in human DAO exhibits 8 amino acid substitutions in pig DAO and 2 amino acid substitutions and a 5 amino acid deletion in rat and mouse DAO, respectively (Fig. 3a). Whereas region P^279^-R^288^ of human DAO has 4 amino acid substitutions in pig DAO, it is identical in rat and mouse DAO except for a single change of H^285^ in human DAO to Y in all other sequences (Fig. 3a).

**Table 3:** Properties of human DAO-specific monoclonal antibodies

Antibody	Isotype	huDAO	piDAO	raDAO	moDAO	IP_hu_	IP_pi_	BR
HYB313-01	IgG2aκ	++	−	−	−	+	−	V^262^-E^278^
HYB313-02	IgG1κ	++	−	−	−	+	−	V^262^-E^278^
HYB313-03	IgG1κ	++	−	−	−	−	−	V^262^-E^278^
HYB313-04	IgG1κ	++	−	−	−	+	−	V^262^-E^278^
HYB311-01	IgG2aκ	++	−	−	−	+	−	P^279^-R^288^

Species cross-reactivity was tested on blots of human (hu), pig (pi), rat (ra), and mouse (mo) kidney or intestine lysates that had comparable DAO enzymatic activity. Immunoprecipitation of DAO was tested with human (IP_hu_) and porcine (IP_pi_) kidney lysates, respectively. The binding region (BR) specifies the peptide of human DAO recognized on blots. ++ strong/+ weak/− no binding or immunoprecipitation

In order to test if the antibodies bind to the native human DAO protein, we performed immunoprecipitation experiments with tissue lysates made in a buffer that preserved the conformation and enzymatic activity of DAO. As shown in Table 3, antibodies HYB313-01/-02/-04 and HYB311-01 weakly immunoprecipitated native human DAO, whereas HYB313-03 did not immunoprecipitate native DAO at all, indicating that the antibodies do not or not efficiently bind to the native protein. This might be explained by the fact that denatured DAO fragments were used for immunizations to obtain these antibodies [12]. Although the peptides identified to be responsible for antibody binding are localized on the surface of the DAO protein (Fig. 3b, c), their conformation in the native protein is probably inadequate for efficient antibody binding.

## Discussion

The binding sites of five monoclonal antibodies specific for human DAO [12] were mapped by binding to blots of DAO peptide fragments expressed as GST fusions in vitro (Fig. 2) and by inhibition of antibody binding to full-length human DAO by pre-incubation with soluble fusion proteins containing small DAO peptides (Table 2). From these experiments, it could be concluded that antibodies HYB313-01/-02/-03/-04 bind in region V^262^-E^278^ and HYB311-01 binds in region P^279^-R^288^ between the N3 and catalytic domains of DAO (Fig. 3). Although we did not exactly map each epitope using synthetic peptides, the binding position information is precise enough and adequate to understand antibody binding characteristics and cross-reactivity. Interestingly, the small region where all antibodies bind was the longest predicted epitope with the highest scores identified by the linear epitope prediction software [23]. It is still surprising that no antibodies had been obtained recognizing other regions of the protein despite using fragments for immunizations covering the complete DAO protein sequence [12].

The antibodies were obtained by immunization of mice with human DAO fragments expressed in vitro that were originally insoluble and were re-solubilized and purified by denaturing SDS polyacrylamide gel electrophoresis [12]. This might explain why the antibodies exhibit excellent binding to denatured DAO on immunoblots and in immunohistochemistry [12] but fail to bind efficiently to the native protein as was evident from the immunoprecipitation results (Table 3). Therefore, these antibodies will not be useful for analysing native human DAO such as in an ELISA but should only be employed in techniques where the protein is denatured. Further, binding occurs in a region of DAO that exhibits considerable sequence variation in different species (Fig. 3a). and therefore, the antibodies can be used only for analyses of the human protein and possibly for other Primates where the sequence of the binding region is almost identical.

Slight differences in the results of experiments testing direct binding to small DAO peptide fragments, binding inhibition by soluble peptides, and immunoprecipitation of native DAO indicated that the epitopes recognized by antibodies HYB313-01/-02/-03/-04 are overlapping but not identical (Fig. 2d; Tables 2, 3). In contrast, the epitope recognized by HYB311-01 is clearly separated and does not overlap the epitopes recognized by the other antibodies (Fig. 2d; Table 2). Having antibodies binding separate non-overlapping epitopes will facilitate the comprehensive analysis of normal human DAO but also of all DAO variants resulting from altered DAO gene sequences. Our binding studies with partial DAO peptide fragments showed that the antibodies will bind to any DAO variant that contains an unaltered binding sequence. The antibodies will be especially useful for the study of SNPs leading to amino acid substitutions associated with altered enzyme function that might be relevant for various human diseases [15–17]. The SNPs with possible clinical relevance described so far including the T^16^M, S^332^F, M^479^I, H^645^D, and N^659^H variants are localized outside the antibody binding region, and the respective altered proteins should therefore be recognized by all our antibodies.

## Abbreviations

DAO: Diamine oxidase
GST: Glutathione *S*-transferase
HDC: Histidine decarboxylase
HNMT: Histamine *N*-methyltransferase
SNP: Single nucleotide polymorphism
